# Role of Cellular Metabolism in the Formation of Neutrophil Extracellular Traps in Airway Diseases

**DOI:** 10.3389/fimmu.2022.850416

**Published:** 2022-04-12

**Authors:** Gabriel Morán, Benjamín Uberti, John Quiroga

**Affiliations:** ^1^Instituto de Farmacología y Morfofisiología, Facultad de Ciencias Veterinarias, Universidad Austral de Chile, Valdivia, Chile; ^2^Instituto de Ciencias Clínicas Veterinarias, Facultad de Ciencias Veterinarias, Universidad Austral de Chile, Valdivia, Chile; ^3^Escuela de Graduados, Facultad de Ciencias Veterinarias, Universidad Austral de Chile, Valdivia, Chile

**Keywords:** neutrophils, NETs, innate immunity, cellular metabolism, airway inflammation

## Abstract

Neutrophil extracellular traps (NETs) are a recently described mechanism of neutrophils that play an important role in health and disease. NETs are an innate defense mechanism that participate in clearance of pathogens, but they may also cause collateral damage in unrelated host tissues. Neutrophil dysregulation and NETosis occur in multiple lung diseases, such as pathogen-induced acute lung injury, pneumonia, chronic obstructive pulmonary disease (COPD), severe asthma, cystic fibrosis, and recently, the novel coronavirus SARS-CoV-2. More recently, research into immunometabolism has surged due to the possibility of reprogramming metabolism in order to modulate immune functions. The present review analyzes the different metabolic pathways associated with NETs formation, and how these impact on pathologies of the airways.

## 1 Introduction

Innate immunity is a primary respiratory defense line against pathogens, allergens and environmental pollutants (1); neutrophils are a major presence in respiratory organs (2). These cells are the first responder cell type for combating pathological insults (3, 4) or sterile lesions, in which they contribute to healing and recovery besides participating in immune responses (5, 6). These cells can release protease and microbicide peptide-containing granules, they produce intra- and extracellular reactive oxygen species (ROS), and they can phagocyte microorganisms or release chromatin traps with cytoplasmic and granular proteins which include myeloperoxidase and elastase (7, 8). Chromatin traps, described in 2004 by using bacteria as stimuli, are termed neutrophil extracellular traps (NETs) (9). However, there is evidence to suggest that other microorganisms besides bacteria (viruses, fungi, protozoa) and non-microbial molecules can also induce NETs release by human neutrophils (8, 10). Unfortunately, pulmonary dysregulation of neutrophil NETosis occur in multipe diseases such as chronic obstructive pulmonary disease (COPD), severe asthma, cystic fibrosis (CF), and the novel coronavirus SARS-CoV-2. In this review, we discuss metabolic reprogramming regarding dysregulation of innate immune responses, focusing on NETs formation in asthma, COPD, CF and SARS-CoV-2.

## 2 Role of Metabolic Reprogramming in Cells

Cellular metabolism can be defined as a set of fundamental biochemical processes through which cells maintain their energy homeostasis and receive the necessary components for the biosynthesis of macromolecules (11). Immune cell metabolism directly influences their differentiation and function, which affects immunity, tolerance, and the inflammatory response (12). Inflammation entails dramatic changes in the metabolism of the affected tissue, including nutrient depletion, increased consumption of molecular oxygen (O_2_), and generation of ROS and reactive nitrogen species (13). These changes in tissue metabolism result, at least partially, from the massive inflammatory cell influx, especially of the myeloid type such as monocytes and neutrophils, which dramatically modify their functional activity in response to proinflammatory agents (5, 14). These environmental and functional alterations represent a major metabolic stress, which is usually efficiently managed due to cells ability to dynamically reprogram their metabolism (15, 16). How cells modify their metabolic patterns to adapt to the inflammatory environment and respond in the face of insult determines the course and prognosis of these diseases (11, 17). If these metabolic processes are dysregulated, the innate immune function can be significantly impaired. Immunometabolism emerges as a new field of research at the interface between immunology and metabolism, historically separated disciplines (17, 18).

## 3 Metabolic Requirements for NET Formation

Formation of NETs, or NETosis, is a cell death mechanism (different from necrosis or apoptosis) which seems to be essential to the innate immune response, permitting capture and degradation of pathogens and their virulence factors (19–21). During NETosis the neutrophil nucleus grows, its chromatin decondenses and long DNA strands are secreted towards the extracellular medium along with cytoplasmic granular proteins (which also disintegrate as the nucleus dissolves) and from chromatin (histones) (9, 22, 23). Bactericidal proteins associated with NETs are mainly cationic and therefore with high affinity to DNA: histones, defensins, proteinase 3, lactoferrin, cathepsin G, neutrophil elastase (NE) and myeloperoxidase (MPO), among others (24). Pentraxin 3 (25) and S100A8/A9 (calprotectin complex, which constitutes 40% of the cytosolic proteins of the neutrophil) are also associated to NETs (24).

**Figure 1:**
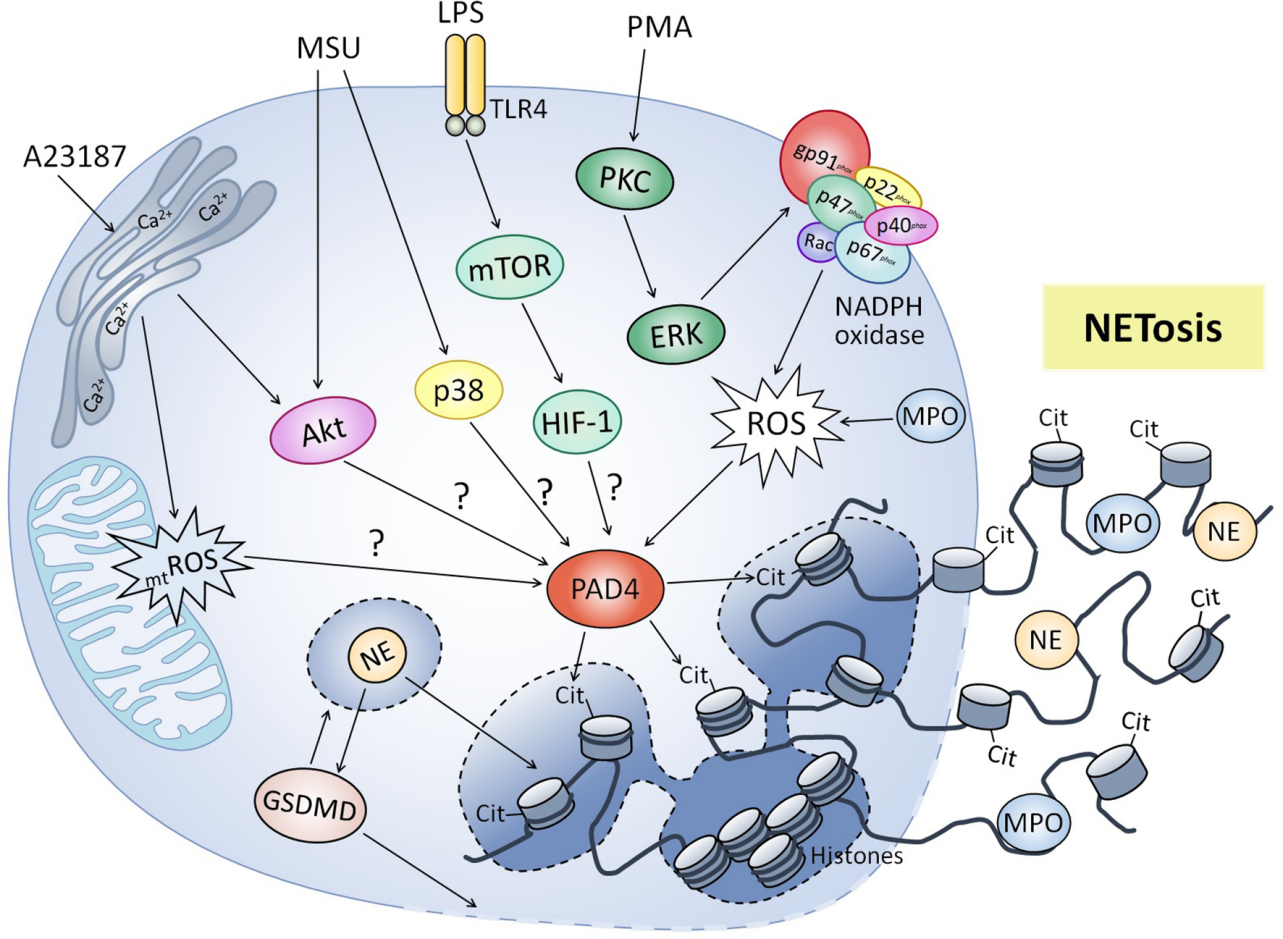
Molecular mechanisms regulating neutrophil extracellular traps formation. Classic NETosis induced by phorbol 12-myristate 13-acetate (PMA) involved activation of NADPH oxidase *via* PKC, production of reactive oxygen species (ROS) and activation of peptidyl arginine deiminase 4 (PAD4), which results in hypercitrullination (cit) of histones and chromatin decondensation. Lipopolysaccharide (LPS) induces NET formation by mTOR/HIF-1 activation, although molecular mechanisms have not been elucidated (?). Other stimuli like calcium ionophore A23187 and monosodium urate (MSU) crystals also trigger NET formation independent of NADPH oxidase, involving signaling pathways like PI3K/Akt and p38 MAPK. Mitochondrial ROS (_mt_ROS) production has been also involved in NADPH-independent NET release. Myeloperoxidase (MPO) participates in NETosis by generating oxidative compounds necessary for the release of neutrophilic elastase (NE). NE also helps chromatin decondensation and activates gasdermin D (GSDMD), which forms pores in the granular and plasma membrane facilitating the release of NETs.

NET formation (**Figure 1**) was initially described and characterized through stimulation of human and murine neutrophils with PMA (26). This classic NETosis mechanism involves NADPH oxidase activation *via* protein kinase C (PKC) and RAF-MEK-ERK pathways, which lead to ROS production and activation of calcium dependent enzyme peptidil arginine deiminase (PAD4), which is particularly abundant in mature neutrophils (27–30). Upon activation, PAD4 translocates to the nucleus and induces histone hypercitrullination, converting positively charged arginine side chains into uncharged histone citrulline side chains, which reduces electrostatic force between histones and DNA and causes chromatin decondensation (31–33). Additionally, MPO converts hydrogen peroxide into hypochlorous acid and other oxidants, which release NE from azurosome in azurophilic granules, allowing its nuclear translocation where it favors chromatin unfolding and nuclear membrane breakdown, releasing chromatin into the cytosol (34–36). NE also cleaves and activates gasdermin D (GSDMD), which leads to pore formation in the granular and plasma membrane, enhancing release of NE and other granular proteases into the cytoplasm, as well as the NE nuclear translocation and GSDMD cleavage (37, 38). Finally, after associating with cytosolic and granular proteins, chromatin is secreted extracellularly (23). Other stimuli apart from PMA can trigger the NETs formation depending on the respiratory burst, including some classical pathogens as bacteria and its products (28, 39–41), viruses (42–44), fungi (45–47) and parasites (48–51).

Otherwise, NET release has been also described independently of NADPH activation after stimulation with agonists as calcium ionophores (52, 53), non-esterified fatty acids (NEFAs) (54), uric acid (55), monosodium-urate (MSU) crystals (56) and pathogen agents as *Staphylococcus aureus* (*S. aureus*) (57), *Candida albicans* (58), *Entamoeba histolytica* (*E. histolytica*) (30) and Dengue virus (59). Interestingly, NET release induced by MSU crystals in human neutrophils (56) and D-lactate in bovine neutrophils (60) was independent of respiratory burst but dependent on PAD4 activity, suggesting that the latter can be also activated by mechanisms independent of NADPH oxidase. NET formation independently of NADPH oxidase and PAD4 induced by *E. histolytica* has also been described in human neutrophils (61), as well as the independently of PAD4 in a murine model of pneumonia caused by *Klebsiella pneumoniae* (62). Neutrophil mitochondria also appear to play a role in NETosis mechanisms. In this respect, NET release induced by the calcium ionophore A23187 in human neutrophils was triggered through a mechanism independent of NADPH oxidase activity but dependent on mitochondrial ROS production (52). Likewise, mitochondria participate in NET formation induced by platelet-activating factor (PAF) in bovine neutrophils by producing ATP necessary to activate purinergic signaling mechanism (63). Supporting the above, ATP contributes to classical PMA-induced NETosis, and NADPH oxidase independent NET formation triggered by A23187 in murine neutrophils (64). Furthermore, some studies have suggested that extracellular DNA strands could have a mitochondrial origin, which would be also compatible with the functional activity of the neutrophil without involving its death (65–69).

In addition to the involvement of RAF-MEK-ERK signaling pathways for NETosis, contribution of the PI3K/Akt axis has also been reported with several stimuli capable of inducing NET formation through the classical pathway (52, 70–72) and NADPH oxidase-independent pathway (30, 52, 56). The PI3K/Akt signaling pathway regulates classic NETosis induced by PMA (52, 70) and immune complexes (71), as well as NADPH oxidase-independent NETosis induced by *E. histolytica* (30), A23187 (52) and MSU crystals (56). In addition, contribution of p38 MAPK in NETosis induced by PMA, MSU crystals, histamine, bacteria and parasites has been demonstrated (28, 29, 49, 56, 72), while TAK1 and Syk pathways in NET formation induced by MSU crystals have also been observed (56). Interestingly, mammalian target of rapamycin (mTOR) mediates LPS-triggered NET formation in murine and human neutrophils by post-transcriptional control of expression of hypoxia-inducible factor 1´s alpha subunit (HIF-1α) (73). Although the mechanisms through which HIF-1 participates in NETs formation aren´t fully known, HIF-1´s role in regulating the expression of enzymes associated with glycolytic metabolism is widely understood (74, 75), suggesting an association between NETosis and metabolism.

**Figure 2:**
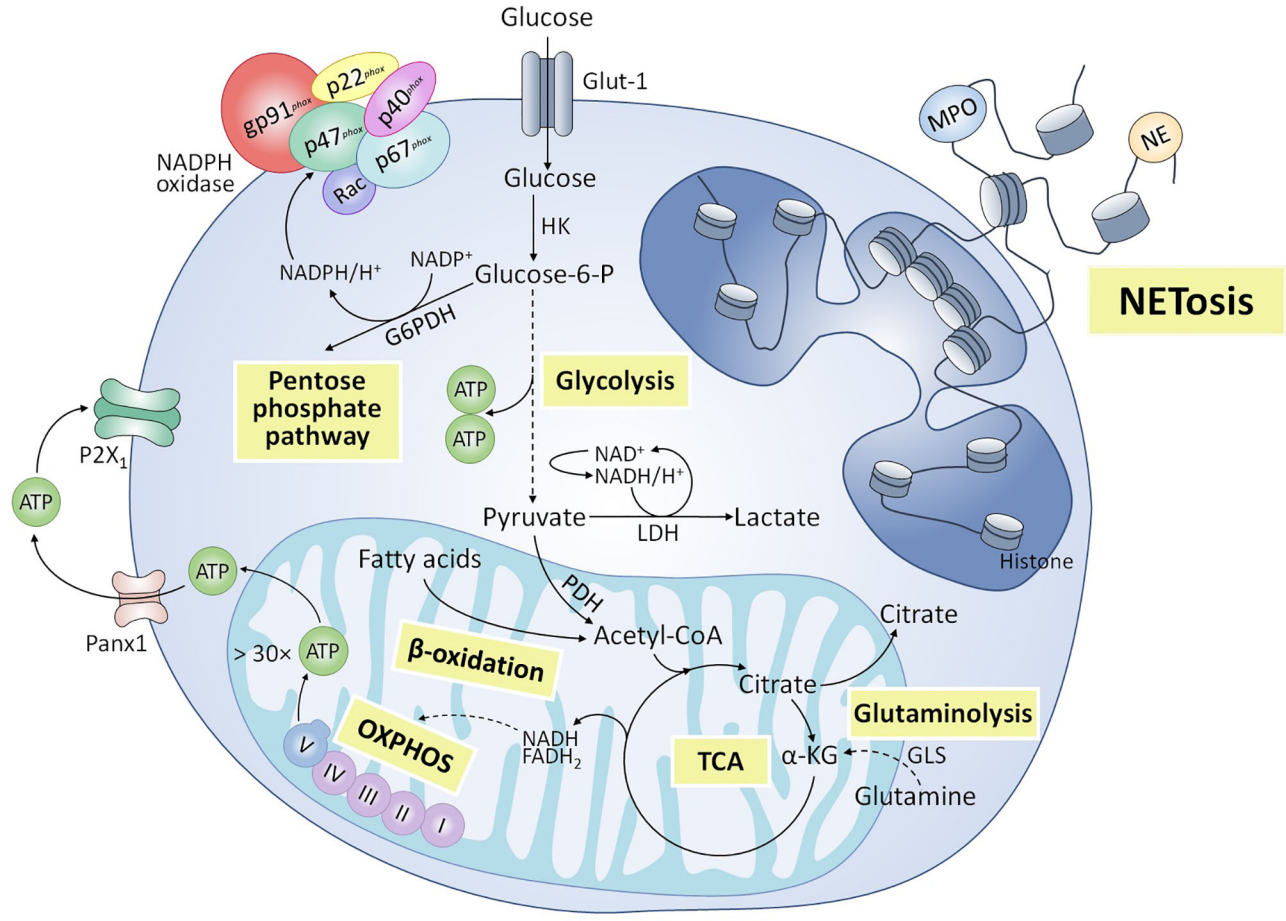
Metabolic pathways involved in NETosis. Various metabolic routes have been implicated in the release of NETs. Glycolysis is an essential metabolic pathway for NADPH oxidase-dependent and -independent NETosis triggered by various stimuli. Pentose phosphate pathway has also been shown to participate in classical NETosis, by providing the NADPH necessary to produce reactive oxygen species (ROS) by the NADPH oxidase complex. Other metabolic pathways associated with mitochondrial activity also appear to be partially involved in NET formation, including hydrolysis of glutamine by glutaminase (GLS) and subsequent anaplerotic reactions to form α-ketoglutarate (α-KG) that enters the tricarboxylic acid (TCA) cycle, and β-oxidation of fatty acids to supply acetyl-coenzyme A (CoA) to the TCA cycle. In addition, the adenosine triphosphate (ATP) synthesized in mitochondria as a product of oxidative phosphorylation (OXPHOS) would be released into the extracellular space through pannexin 1 (Panx1) channels and would enhance NET formation through purinergic signaling mechanisms dependent on P2X_1_ receptors.

Metabolic requirements (**Figure 2**) for NET formation have been an object of study in the past years, with glycolysis identified as a pivotal metabolic pathway for its development. Initially, Rodríguez-Espinosa et al. demonstrated that NETosis induced by PMA in human neutrophils depends on exogenous glucose and glutamine (26). Furthermore, glycolysis inhibition through 2-deoxy-D-glucose (2-DG) blocks PMA-triggered NET release, while the ATP synthase inhibitor oligomycin only partially reduces it (26). Similar relevance to exogenous glucose and glycolysis was demonstrated by Azevedo et al. in classical NETosis triggered by PMA and amyloid fibrils in humans, further identifying a key role of the pentose phosphate pathway, since glucose-6-phosphate dehydrogenase provides NADPH to NADPH oxidase, for ROS production and NETs release (76). Amini et al. also reported a decrease in NETosis induced by granulocyte-macrophage colony-stimulating factor (GM-CSF) + C5a due to glycolysis inhibition by 2-DG in human and murine neutrophils (77). However, mitochondrial complex I activity was also involved in NET release (77). More recently, Quiroga et al. also showed that blocking glycolysis with 2-DG inhibited NETosis triggered by PAF in bovine neutrophils (63). In addition, blocking mitochondrial complex I activity and oxidative phosphorylation with rotenone and carbonyl cyanide 3-chlorophenylhydrazone (CCCP), respectively, prevented extracellular ATP release and NETosis, and pharmacological inhibition of P2X1 purinergic receptor inhibited NET formation, suggesting the role of purinergic signaling in NETosis mechanism (63). In contrast to the above, Zhou et al. observed an increase in glucose consumption after *Besnoitia besnoiti* tachyzoite exposure in bovine neutrophils; however, glycolysis blocking by 2-fluor-2-deoxy-D-glucose (FDG) didn´t influence NET release (78). Interestingly, these authors also observed a relevant role of mitochondrial ATP and P2X1 receptor-dependent purinergic signaling for tachyzoite-triggered NETosis (78). In agreement with previous authors, Alarcón et al. recently showed that NET formation induced by NEFAs in bovine neutrophils was strongly dependent on purinergic signaling, since pharmacological inhibition of pannexin 1 channels and P2X1 receptors reduced NET release partially and totally, respectively (54). In addition, inhibition of β-oxidation with etomoxir partially reduced NETosis induced by NEFAs, suggesting some degree of involvement of this metabolic pathway (54).

The molecular mechanisms involved in the NET formation, as well as the metabolic pathways on which it depends, seem to be mainly dependent on the inducing stimulus and the inflammatory context. However, they are not yet fully understood and are still being studied.

## 4 Metabolic Adaptations in Neutrophils During Airways Pathological Conditions

### 4.1 Cystic Fibrosis (CF)

CF is a monogenic multiorganic disease that affects epithelial organs, with mortality often occurring from airway disease (79). Pulmonary damage is a consequence of chronic airway inflammation associated with bacterial colonization (80). In the disease, the CF transmembrane conductance regulator (CFTR) gene, encoding a chloride-bicarbonate transmembrane anion channel, suffers autosomal recessive mutations (81). In this disease, NETs have a deleterious effect in inflammation and lung destruction instead of working in their antimicrobial capacity. Some studies have shown that extracellular DNA concentration correlates with lung neutrophil concentration, and therefore can be used to measure pulmonary inflammation and severity of disease (82, 83). Neutrophils fail to clear infections in cases of CF, due to reduction of CD16-CD14 receptors and functional changes (84). The same authors observed an increase in lipid raft assembly, granule mobilization and CD11b and CD66 expression. Studies on metabolic reprogramming in CF airway neutrophils may explain how such dysfunctional phenotype develops. The mTOR pathway is activated in CF airway neutrophils (85), and glucose transporter 1 (Glut1) and inorganic phosphate transporter 1 (PiT1) expression both increase (86), all of which promotes glucose utilization in the airways (87). Other authors have also shown that airway neutrophils in CF patients respond to excess free glucose and amino acids, concomitantly increasing Glut1 expression. CF cases also increase production of resistin, a regulatory protein (88) closely related to insulin resistance (89). While insulin resistance impairs glucose uptake by cells, anabolic reprogramming of CF airway neutrophils allows them to efficiently uptake and utilize glucose, fueling their pro-survival pathways (84, 85). Unfortunately, resistin also diminishes neutrophils´ bactericidal ability because it inhibits actin polymerization and ROS production, as has been observed for the pathogens *Pseudomonas aeruginosa* and *S. aureus* which are closely associated to CF (90). On the other hand, the mechanisms that regulate NET formation, especially during chronic lung inflammation, as occurs in CF, are not well understood. One study showed that the G protein-coupled receptor (GPCR) CXCR2 mediates NET formation (91) independently of NADPH oxidase, but with involvement of Src family kinases. Pulmonary blockade of CXCR2 inhibits NET formation and ameliorated lung function *in vivo*, with no effect on neutrophil recruitment, proteolytic activity or antibacterial defense of the host. Those authors concluded that CXCR2 is a receptor that mediates NET formation independently of NADPH oxidase (91).

### 4.2 Chronic Obstructive Pulmonary Disease (COPD)

Chronic obstructive pulmonary disease (COPD) is a leading cause of worldwide morbidity and mortality (92). Chronic exposure to cigarette smoke, environmental pollutants or other inhaled irritants, is its primary cause (93). Neutrophils correlate directly with severity and inflammation and seem to be key effectors in the pathophysiology of the disease (94, 95). Progressive destruction of lung parenchyma, as well as poor responses in infective exacerbations, are thought to be due to dysregulated functions of neutrophils in COPD patients, which has been proven *in vitro* (96). NETs formation is increased in the sputum of stable COPD patients, which correlates with airway neutrophil numbers and extracellular DNA concentration, limited lung function and disease severity (97–99). Likewise, expression of PAD4 gene is up-regulated in neutrophilic COPD cases, compared to non-neutrophilic patients (100). Increased expression of PAD4 has been described in lung samples from COPD patients (101), which leads some authors to support that NETs formation could be a feature of the NET-COPD phenotype (102). Furthermore, peripheral blood neutrophils from e-cigarette users had a high susceptibility to NETosis induction, and there were more NETs-related proteins in these subjects´ sputum compared to nonsmokers (103).

Although glycolysis is the primary energy source for neutrophils, their few mitochondria are functional and contribute to ATP and ROS production, which are necessary for migration (104, 105). Some authors suggest that alterations of mitochondrial function and impaired glucose metabolism both may lead to defects in neutrophil migration and function in cases of COPD (1). Of the latter, one study demonstrated elevated glucose levels in the sputum of COPD patients, and this glucose increased further in response to exacerbations or experimental rhinovirus infection. Furthermore, they showed that increased glucose concentration in sputum samples could support bacterial growth, suggesting that increased glucose in the airways in response to viral infection could in turn facilitate secondary bacterial infections (106). However, in this study the production of NETs was not quantified, so we could not make an association between sputum glucose and NET formation, but as glycolysis is important in the production of this phenomenon, we could infer that there is an alteration of neutrophil cellular metabolism in COPD patients. Ultimately, the mechanisms of NET formation in COPD are still being explored and more studies are needed to understand this phenomenon. Some authors claim that it is unclear whether neutrophils undergo NETosis after migration into the lungs, or rather, whether they are constitutively primed to undergo this response during COPD-related inflammation while still in circulation (107). Cellular metabolic changes could also be important in defective innate immune responses in COPD, and unraveling these neutrophilic metabolic changes could lead to novel unconventional therapies for this pathology.

### 4.3 Severe Asthma

Asthma is a chronic inflammatory airway disease of enormous importance, with approximately 350 million cases around the world (108). It represents 1.1% of total global disease and produces loss of 26.2 million disability-adjusted life years according to WHO (109). The disease is generally characterized by airway inflammation, hyperresponsiveness, and reversible airflow obstruction (110, 111). The type and degree of airway inflammation and airway remodeling are heterogeneous, as are symptoms. Thus, various phenotype models have been developed (112), representing observable clinical characteristics which include age of onset (early or late onset), possible comorbidities (obesity and others), exacerbating factors (infections, allergens, and exercise among others), and response to treatment (for example, response to steroids) (113). Airway infiltration with eosinophils and mast cells -and their role in disease progression- is well described, and neutrophil infiltration has also been assessed in many clinical studies and is associated with disease severity (114). Marked relative neutrophilia in the sputum (more than 60%) is a more frequent finding in older adults. These patients are predominantly male, develop late-onset asthma, have more severe lung disease, resistance to treatment with corticosteroids, and a higher risk of hospitalization (115). They also had more comorbidities, including hypertension, osteoporosis, gastro-esophageal reflux disease, smoking, and obesity (115–118).

Neutrophils and NET secretion are fundamental in the pathogenesis of early asthma according to murine models of asthma (119–122). However, evidence for NETs in the pathogenesis of early asthma in human patients is still scarce. Peripheral blood NETs concentration is higher in children with asthma, particularly during episodes of symptom exacerbation, and studies showed that asthmatic children´s neutrophils can produce more NETs than healthy controls *in vitro* (123). In addition, extracellular DNA was higher in severe asthma cases´ sputum samples than in those of mild to moderate asthma (100). In that study, NETs levels were not only higher in asthmatic patients than in healthy controls: extracellular DNA concentration also correlated with asthma severity. IL-1β, IL-8 and gene expression levels of inflammasome components (such as NLRP3) sputum concentrations were also higher in patients with elevated extracellular DNA concentrations. Other authors also reported high concentrations of extracellular DNA in the sputum of severely asthmatic patients, with airway NETs and markers of inflammasome activation (124). However, as demonstrated in another study, circulating NETs seem to be better indicators of asthma severity than alveolar NETs concentration (125). Therefore, further clinical investigations are needed to elucidate the role of NETs in the progression of asthma. Nevertheless, studies have shown a cytotoxic effect of NETs in lung epithelial and endothelial cells (126–128). NETs can also impair lung epithelial barrier functions (129), induce release of proinflammatory mediators from dendritic cells, and of proteases from neutrophils (130). Authors suggest that NETs may act directly on airway epithelial cells, inducing secretion of inflammatory factors, aggravating airway inflammation and worsening respiratory symptoms (124, 126). Other studies have also shown that the protease contained in NETs activates proinflammatory cytokines, thus aggravating the inflammatory response (131). Thus, all these studies suggest that NETs destroy the integrity of the airway epithelium, increase cytokine secretion, and lead to asthma progression.

Several investigations relate metabolomic profiles to human asthma, focusing on metabolites as systemic biomarkers (132–135). Metabolites have also been studied in an equine model of asthma (136, 137), with neutrophilic phenotypic characteristics (138, 139). Previous studies have characterized the metabolic profile of circulating neutrophils, but those of pulmonary neutrophils are few. However, similar to severe asthma in humans, airway neutrophilia is typical of asthmatic horses (138, 139). NETs and evidence of oxidative stress have been described in bronchoalveolar lavage fluid (BALF) of asthma-affected horses (140). Albornoz et al. (137) found increased lactate, citrate and arabitol in BALF of asthmatic horses, suggesting elevated energy demand during exacerbation of the disease. This lactate increase has also been previously reported both in asthmatic children´s urine (141) and in BALF from patients with CF (142), demonstrating that those respiratory diseases course with similar energetic metabolic changes. In turn, an elevated citrate concentration was observed in horses with equine asthma (137), which could be due to an increase in glycolysis energy flow and alterations in Krebs cycle function, which can occur in activated inflammatory cells (143). Similarly, an increase in arabitol accumulation was observed in equine asthma (137). These same authors argue that this increase in arabitol could be explained by an increase in the oxidative phase of pentose phosphate pathway originating from large quantities of neutrophils recruited to the inflamed airways. However, the contribution of other cell types to metabolomic changes in BALF cannot be ruled out.

### 4.4 Severe Acute Respiratory Syndrome Coronavirus 2 (SARS-CoV-2)

Severe acute respiratory syndrome coronavirus (SARS-CoV)-2 was first found in Wuhan, China (144). The worldwide pandemic of coronavirus disease 19 (COVID-19) has affected all of humanity, with millions of deaths. Although SARS-CoV-2 can extend to many organs, including the heart, kidneys, gut, blood vessels and brain, it is initially a pulmonary disease (145). Viral replication and its subsequent immune response both contribute to COVID-19´s severity: in some patients, this entails a cytokine storm and severe inflammatory response syndrome (SIRS), secondary sepsis, multiorganic failure, and eventually death (146). Since the start of the COVID-19 pandemic, progressive pathophysiologic evidence shows that neutrophils play an important role in the disease, especially in severe patients (147). Neutrophil defense mechanisms (phagocytosis, ROS, NETosis among others) are differentially active in neutrophil subsets. The capacity of these mechanisms increases with maturation phase (148, 149). In circulation, progressive granulation in aging neutrophils impairs their functions and NET formation (150). The importance of this last sentence lies in the fact that in severe SARS-CoV-2 infection it is marked by altered abundance, phenotype and functionality of neutrophils. High numbers of neutrophils have been found in the nasopharyngeal epithelium, distal lung and in blood counts (151–153). Likewise, studies show changes in gene expression in blood neutrophils with pre-mature phenotypic markers in severe cases of COVID-19 (154, 155).

Analysis of COVID-19 patient lung samples showed neutrophilic mucositis, neutrophil infiltration of pulmonary capillaries, fibrin deposition, and neutrophil extravasation of neutrophils towards the alveolar space (156). Fox et al. (157) observed neutrophils and platelets trapped in a fibrin mesh within the alveolar capillaries of patients with COVID-19, which suggests the presence of NETs (157). In turn, other authors have detected augmented serum cell-free DNA concentration, DNA-MPO complexes, and citrullinated histone H3 (Cit-H3), all of which correlate with disease severity (158). In fact, through unknown mechanisms, viable SARS-CoV-2 can directly induce NET formation in healthy neutrophils (159). Likewise, NET formation and alveolar epithelial necrotic cell death both release damage-associated molecular patterns which entail production of proinflammatory cytokines, and vice versa, establishing a necroinflammatory loop that is responsible for cytokine storm and sepsis (160). There are still no studies of alterations in neutrophil cellular metabolism for the induction of NETs, but there are metabolomic studies showing that increased glucose concentration and glycolysis promote cytokine production by monocytes and replication of SARS-CoV-2 (CoV-2) *via* a mitochondrial ROS/HIF-1α dependent mechanism, which lead to T-cell and epithelial cell death. Therefore, people with hyperglycemia (for example, diabetics) have an increased risk of developing severe COVID-19 disease, which suggests that metabolism plays an important role in the disease progression of COVID-19 infection (161, 162). Of the latter, it is known that the tricarboxylic acids (TCA) cycle produces metabolites used in the synthesis of amino acids, lipids and nucleotides, all of which viruses need to replicate (163). In addition, mTOR complex 1 (mTORC1) has been shown to regulate mitochondrial activity and the anabolic metabolism, while on the contrary, mTORC1 inhibitors can decrease TCA cycle metabolite concentrations (164). Mullen et al. (165) suggest that SARS-CoV-2 readjusts carbon input into the TCA cycle, which results in a reduction of the oxidative metabolism of glutamine and increases the input of pyruvate, *via* pyruvate carboxylase. These same authors demonstrated that inhibition of mTORC1 produces a decrease in SARS-CoV-2 infection, which could justify a potential therapeutic option for treating patients with COVID-19; however, further studies are needed to understand the mechanism of inhibition and its potential applications and efficacy in patients.

## 5 Conclusion

Recent advances in the field of immunometabolism emphasize the plasticity of neutrophil biology in health and disease. In addition, neutrophil-mediated innate immunity has been redefined since the identification of NETs. NETosis plays an important role in physiological and pathological conditions, and their metabolic implications (**Table 1**) must be understood in order to enable exploration of possible therapeutic interventions. Although formation of NETs is in essence a useful antimicrobial defense strategy, dysregulation of the process may entail tissular adverse effects and hence contribute to NETopathic lung inflammation. Novel therapeutic strategies targeted at neutrophils, such as inhibitors of neutrophil recruitment or NET formation, may help reduce the severity of multiple pulmonary diseases, including asthma, COPD, CF and COVID-19.

**Table 1:** Molecular mechanisms and metabolic pathways possibly altered in leucocytes in airway diseases.

Airway disease	Molecular mechanisms	Metabolic and functional consequences	References
Cystic fibrosis (CF)	Decreased expression of CD16 and CD14 receptors, mobilization of CD63+ NE-rich granules, lower levels of glutathione, expression of CD80, MHC type II and CD294	Neutrophils dysfunctional phenotype	(84)
	Elevated mTOR pathway signaling	Increased glucose utilization	(85)
	Increased expression of Glut1 and PiT1	Increased glucose utilization	(86, 87)
	Increased production of resistin	Decreased ROS production and actin polymerization	(88, 90)
	CXCR2 signaling	NADPH oxidase independent NETosis	(91)
Chronic obstructive pulmonary disease (COPD)	Upregulation of PAD4	Increased NET formation	(100, 101)
	Impaired mitochondrial function and glucose metabolism	Defective neutrophil migration and function	(1)
Severe asthma	Increase in energy flow through glycolysis and alterations in TCA	Increase in lactate and citrate levels	(137)
	Increase in oxidative phase of pentose phosphate pathway	Increase in arabitol	(137)
Severe acute respiratory syndrome coronavirus 2 (SARS-CoV-2)	Elevated glucose and glycolysis	Increased viral replication and cytokine production *via*_mt_ROS/HIF-1α dependent mechanism	(161)
	Carbon input readjustment into TCA cycle	Increased input of pyruvate and reduced glutamine oxidation	(165)

## Author Contributions

All listed authors have made a direct and substantial contribution to this work and approved the final manuscript.

## Funding

This work was partially supported by FONDECYT grant 1190369.

## Conflict of Interest

The authors declare that the research was conducted in the absence of any commercial or financial relationships that could be construed as a potential conflict of interest.

## Publisher’s Note

All claims expressed in this article are solely those of the authors and do not necessarily represent those of their affiliated organizations, or those of the publisher, the editors and the reviewers. Any product that may be evaluated in this article, or claim that may be made by its manufacturer, is not guaranteed or endorsed by the publisher.
